# Transcutaneous auricular vagus nerve stimulation for paroxysmal sympathetic hyperactivity syndrome after intracerebral hemorrhage: a hypothesis-generating case report

**DOI:** 10.3389/fnins.2026.1828720

**Published:** 2026-04-23

**Authors:** Xinyuan Han, Zhijun Huang, Ziting Gao, Yangyang Feng, Yu Li

**Affiliations:** Department of Neurological Rehabilitation, Shaanxi Provincial Rehabilitation Hospital, Xi'an, China

**Keywords:** case report, cerebral hemorrhage, neuromodulation, paroxysmal sympathetic hyperactivity, tachypnea, transcutaneous auricular vagus nerve stimulation

## Abstract

**Objective:**

To observe the clinical effect of transcutaneous auricular vagus nerve stimulation (taVNS) in drug-refractory paroxysmal sympathetic hyperactivity (PSH).

**Methods:**

This case report describes the clinical course of a 63-year-old male with PSH following intracerebral hemorrhage. PSH episodes were characterized by tachypnea, tachycardia, hypertension, and increased muscle tone. After 2 weeks of combination pharmacotherapy (propranolol, baclofen, gabapentin), blood pressure, heart rate, and muscle tone improved, but tachypnea remained inadequately controlled. Although sedative agents alleviated tachypnea, they led to decreased consciousness level and could not be continued. Subsequently, taVNS was added to the ongoing pharmacotherapy.

**Results:**

After 4 weeks of taVNS treatment, PSH episode frequency and tachypnea improved. Baclofen and gabapentin were discontinued, propranolol dosage was reduced, and the patient’s consciousness level showed improvement. After another 4 weeks of continued treatment, only mild tachypnea occurred occasionally under strong stimulation, without other sympathetic symptoms. Consciousness level further improved.

**Conclusion:**

This case suggests that taVNS may be a safe adjunctive intervention option for drug-refractory PSH. The symptom relief and consciousness improvement observed during treatment may be related to the application of taVNS.

## Introduction

Paroxysmal sympathetic hyperactivity (PSH) is a serious complication after brain injury. Clinical evidence indicates that PSH may be an independent risk factor for poor neurological outcomes (Nasa et al., 2024; Meyfroidt et al., 2017; Yang et al., 2025). Current treatment for PSH primarily relies on pharmacotherapy. However, some patients show poor response, or its clinical use is limited by adverse effects such as excessive sedation and decreased consciousness level (Tu et al., 2021). Transcutaneous auricular vagus nerve stimulation (taVNS) is a non-invasive neuromodulation technique. Preclinical studies suggest that taVNS may have potential therapeutic value for various autonomic nervous system disorders through mechanisms such as activating the cholinergic anti-inflammatory pathway and enhancing parasympathetic tone (Lu et al., 2017; Jiang et al., 2015). This report describes a 63-year-old patient with PSH following intracerebral hemorrhage. After multiple medications showed limited efficacy or could not be continued due to worsening consciousness, combined treatment with taVNS was attempted. This report presents the clinical course and preliminary observation of taVNS for drug-refractory PSH.

## Case description

**Figure 1:**
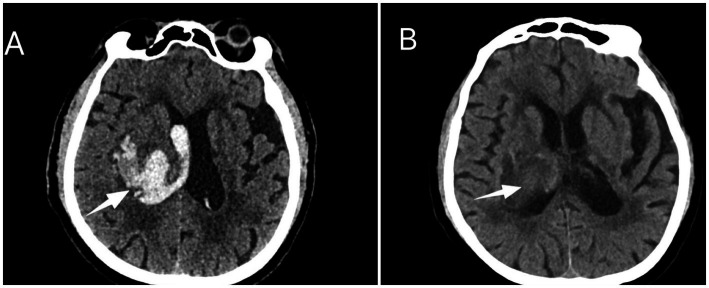
**(A)** Cranial computed tomography demonstrates a patchy hyperdense shadow in the right thalamus, basal ganglia, and paraventricular region. The lesion measures approximately 3.7 × 3.5 × 3.1 cm at its largest cross-section, with a mean density of approximately 67 Hounsfield Units. It is surrounded by a hypodense rim and exerts mass effect on the adjacent lateral ventricle. Hyperdense material is also observed filling the ventricular system. **(B)** Cranial computed tomography reveals a patchy mixed-density shadow, containing both hyperdense and hypodense components, located in the right thalamus, right basal ganglia, and paraventricular area adjacent to the right lateral ventricular body. Mild compression of the right lateral ventricle is noted.

A 63-year-old man was admitted due to sudden altered sensorium for 2 h. He had a 10-year history of hypertension, with blood pressure up to 180/100 mmHg despite regular treatment. On admission, body temperature was 36.6 °C, blood pressure 190/105 mmHg, pulse 110 beats/min, respiratory rate 27 breaths/min, and peripheral oxygen saturation 88–94%. Neurological examination revealed dilated pupils (4 mm), positive left Babinski sign, negative meningeal irritation signs, and a Glasgow Coma Scale score of 5(E1V1M3). The patient was diagnosed with right thalamic, basal ganglia, and lateral ventricular intracerebral hemorrhage (approximately 21 mL) with intraventricular extension on emergent head CT (Figure 1A). The patient was admitted to the neurosurgery intensive care unit. Initial treatment included: intravenous urapidil (2–10 mg/min) for blood pressure control (switched to oral nifedipine 10 mg q8h after 7 days); 20% mannitol (250 mL q12h, tapered regimen) for intracranial pressure reduction; intermittent sedation with midazolam (0.02–0.2 mg/kg/h); and oxygen therapy, cardiac monitoring, and endotracheal intubation. The patient did not undergo decompressive craniectomy or any other decompressive surgery. During treatment, hospital-acquired pneumonia developed, presenting with high fever (39.3 °C) and elevated inflammatory markers (white blood cell count 18.2 × 10^9^/L, neutrophils 91%, procalcitonin 2.6 ng/mL). Sputum culture revealed mixed infection with carbapenem-resistant *Klebsiella pneumoniae* (CR-KP) and methicillin-resistant *Staphylococcus aureus* (MRSA). Susceptibility testing showed: CR-KP resistant to meropenem and sensitive to polymyxin B; MRSA sensitive to linezolid. Targeted anti-infective therapy with meropenem (2.0 g q8h) and linezolid (600 mg q12h) was administered for 14 days, and the infection was controlled. During this period, the patient received mechanical ventilation and nutritional support.

On day 21, after pneumonia improved, the patient was transferred from the neurosurgery intensive care unit to the neurorehabilitation department. At rest, vital signs were stable: heart rate 70–80 beats/min, respiratory rate 14–23 breaths/min, peripheral oxygen saturation 96–100%, blood pressure 120/75 mmHg (maintained with nifedipine 10 mg q8h). The patient was in a comatose state, with a Coma Recovery Scale-Revised (CRS-R) score of 7. Nasogastric tube and urinary catheter were in place. Head CT showed patchy mixed-density areas in the right thalamus, basal ganglia, and periventricular region, with mild compression of the right lateral ventricle (Figure 1B). No obvious signs of infection were present. However, mild stimulation (e.g., turning, passive range of motion, and other routine nursing care) could induce sympathetic episodes characterized by: heart rate rising to 106–145 beats/min, respiratory rate 28–50 breaths/min, peripheral oxygen saturation 90–94%, blood pressure increasing to 150–185/90–115 mmHg, accompanied by increased limb muscle tone, sweating, and temperature fluctuations (36.4–38.2 °C). Each episode lasted from several minutes to 1 h, occurred several to more than 10 times daily, and resolved spontaneously. Most episodes were triggered by mild stimulation, and a few occurred spontaneously. These typical PSH symptoms first appeared around week 3 after intracerebral hemorrhage. Before that, during the stay in the neurosurgery intensive care unit, deep sedation may have masked PSH manifestations. After electroencephalography excluded seizures, according to the PSH Assessment Measure (PSH-AM), the patient’s Clinical Feature Score was 12 (≥8), the Diagnostic Likelihood Tool score was 10 (≥8), and the total score was 22 (≥17). A diagnosis of paroxysmal sympathetic hyperactivity (PSH) was confirmed.

**Figure 2:**
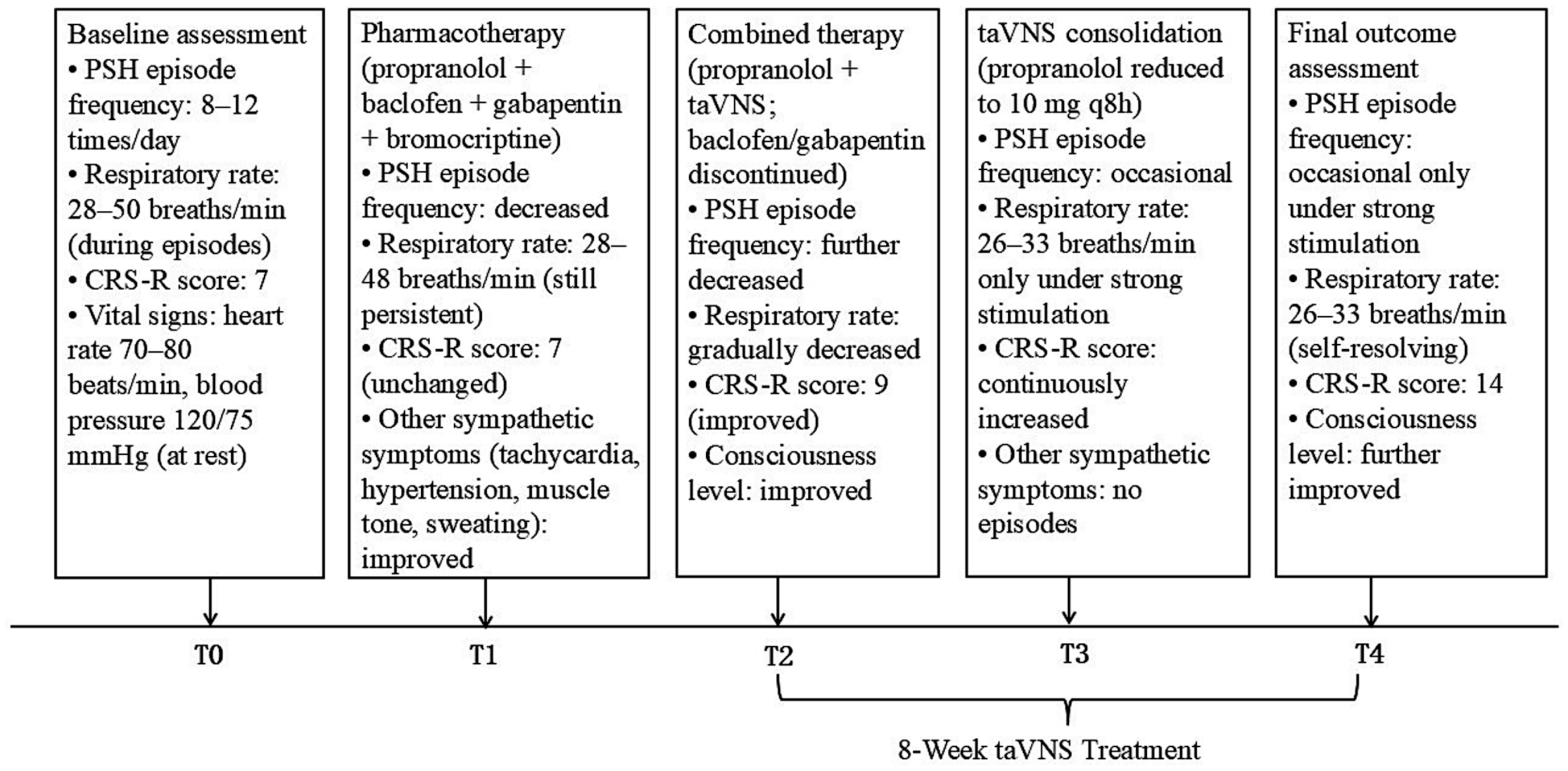
Treatment protocol and clinical course. T0: baseline at PSH diagnosis; T1: after 2 weeks of pharmacotherapy (propranolol, baclofen, gabapentin, bromocriptine); T2: after 4 weeks of combined pharmacotherapy and taVNS (baclofen and gabapentin discontinued, propranolol reduced); T3: after 4 weeks of taVNS consolidation with medication tapering (propranolol 10 mg q8h); T4: final assessment after 8-week taVNS course. CRS-R, Coma Recovery Scale–Revised.

Based on routine rehabilitation, the patient first received propranolol 10 mg q8h, baclofen 10 mg q8h, gabapentin 300 mg q8h, and bromocriptine 2.5 mg q12h as a four-drug combination for 2 weeks. PSH episode frequency decreased. During episodes, tachycardia, hypertension, increased muscle tone, sweating, and fever improved. However, the predominant symptom—tachypnea— showed no obvious relief, with respiratory rate 28–48 breaths/min. To address the persistent tachypnea, propranolol was increased to 20 mg q8h. Heart rate and blood pressure decreased slightly. Respiratory rate remained 29–46 breaths/min. As tachypnea remained poorly controlled, clonazepam (0.5 mg tid) and dexmedetomidine (0.2 μg/kg/h) were added sequentially. It should be noted that after 2 weeks of initial pharmacotherapy, when other PSH symptoms had improved but tachypnea persisted, other potential causes of tachypnea were ruled out. The patient did not have a tracheostomy. Hospital-acquired pneumonia had fully resolved, with normal inflammatory markers and improved chest imaging. Arterial blood gas analysis excluded acidosis. Normal body temperature excluded fever as a cause. Echocardiography and B-type natriuretic peptide excluded cardiac causes. Chest CT and oxygen saturation excluded pulmonary causes. In summary, the tachypnea was determined to be of autonomic origin due to PSH. Key clinical events, medical and nursing interventions are summarized in Table 1. Episode frequency and respiratory rate decreased (12–22 breaths/min). However, consciousness level declined (CRS-R decreased to 3). It should be noted that the decreased level of consciousness was considered primarily related to deep sedation (clonazepam and dexmedetomidine). Head CT showed no cerebral edema or rebleeding. Laboratory tests showed no active infection or other organic causes. To avoid excessive sedation, these agents were discontinued. After discussion with the family and informed consent, taVNS was finally added while continuing propranolol 20 mg q8h, baclofen 10 mg q8h, and gabapentin 300 mg q8h. The treatment protocol is shown in Figure 2.

**Table 1:** Timeline of key clinical events, medical and nursing interventions.

Time phase	Key clinical events and issues	Medical interventions	Nursing assessment and interventions	Outcomes and responses
Acute Phase (Admission)	Intracerebral hemorrhage with unstable vital signs and impaired consciousness.	Blood pressure control, intracranial pressure reduction, sedation, anti-infection therapy.	Nursing Assessment: Continuous monitoring of vital signs, consciousness, and pupils. Nursing Interventions: Medication administration per order, pressure injury prevention, artificial airway management, initiation of early rehabilitation positioning.	Vital signs initially stabilized; hospital-acquired pneumonia occurred.
~3 Weeks Post-Onset (PSH Diagnosis Phase)	Infection controlled, but minimal stimuli triggered PSH episodes (prominent tachypnea).	PSH diagnosis confirmed; initiation of combined pharmacotherapy.	Nursing Assessment: Identification and documentation of PSH triggers, frequency, and manifestations (respiration, heart rate, BP, etc.). Nursing Interventions: Implementation of PSH preventative care (minimizing auditory/light stimuli, clustering care, private room).	Other PSH symptoms improved, but tachypnea persisted.
2 Weeks Post-Pharmacotherapy (End of T1)	Tachypnea became the refractory core problem.	Trial of intensified sedation (clonazepam, dexmedetomidine).	Nursing Assessment: Close monitoring of sedation depth and impact on consciousness. Nursing Interventions: Sedation protocol administration and benefit–risk evaluation. Nursing Finding: Reported significant decrease in consciousness level (CRS-R = 3) due to sedation.	Sedation controlled respiration but was discontinued due to profound consciousness suppression, highlighting therapeutic dilemma.
Initiation of taVNS (Start of T2)	Seeking non-sedative alternative.	Introduction of taVNS while maintaining prior medications.	Nursing Assessment: Evaluation of taVNS tolerance (skin, subjective response). Nursing Interventions: taVNS administration, accurate recording of treatment parameters/duration, and observation of episode frequency changes.	Gradual improvement in PSH episodes and tachypnea noted.
4 Weeks Post-taVNS (End of T2)	Significant symptom improvement and consciousness recovery.	Discontinuation of baclofen and gabapentin; reduction of propranolol dosage.	Nursing Assessment: Monitoring for stability during medication taper. Nursing Interventions: Enhanced rehabilitation and arousal stimulation; documentation of CRS-R score changes.	Achieved symptom control and medication simplification; consciousness improved (CRS-R = 9).
8 Weeks Post-taVNS (T4 / Follow-up)	Condition stabilized with marked consciousness recovery.	Completion of taVNS course.	Nursing Assessment: Comprehensive pre-discharge assessment. Nursing Interventions: Family education on key care points and home rehabilitation planning.	Achieved stable outcome with substantial symptom control and consciousness improvement (CRS-R = 14).

T0, Baseline; T1, Pharmacotherapy phase; T2, Combined pharmacotherapy and taVNS phase; T3, taVNS consolidation with medication tapering; T4, Final assessment after the 8-week taVNS course. Data in the “Outcomes” column are presented as mean (range), derived from repeated daily measurements within each phase. CRS-R, Coma Recovery Scale–Revised.

A taVNS device (Jiangxi Jingyi Medical Technology Co., China) was used. The stimulation site was the right auricular concha (Zanello et al., 2025). Parameters were set as follows: biphasic square wave, pulse width 250 μs, frequency 30 Hz. Stimulation intensity was individually adjusted based on the patient’s blink response, starting at 0.5 mA and gradually increased to 1.0 mA after 1 week (Thompson et al., 2021). An intermittent stimulation mode was used (10 s on, 20 s off). Each session lasted 30 min. Treatment frequency was twice daily, 5 days per week, and was adjusted to once daily after 4 weeks.

## Results

**Figure 3:**
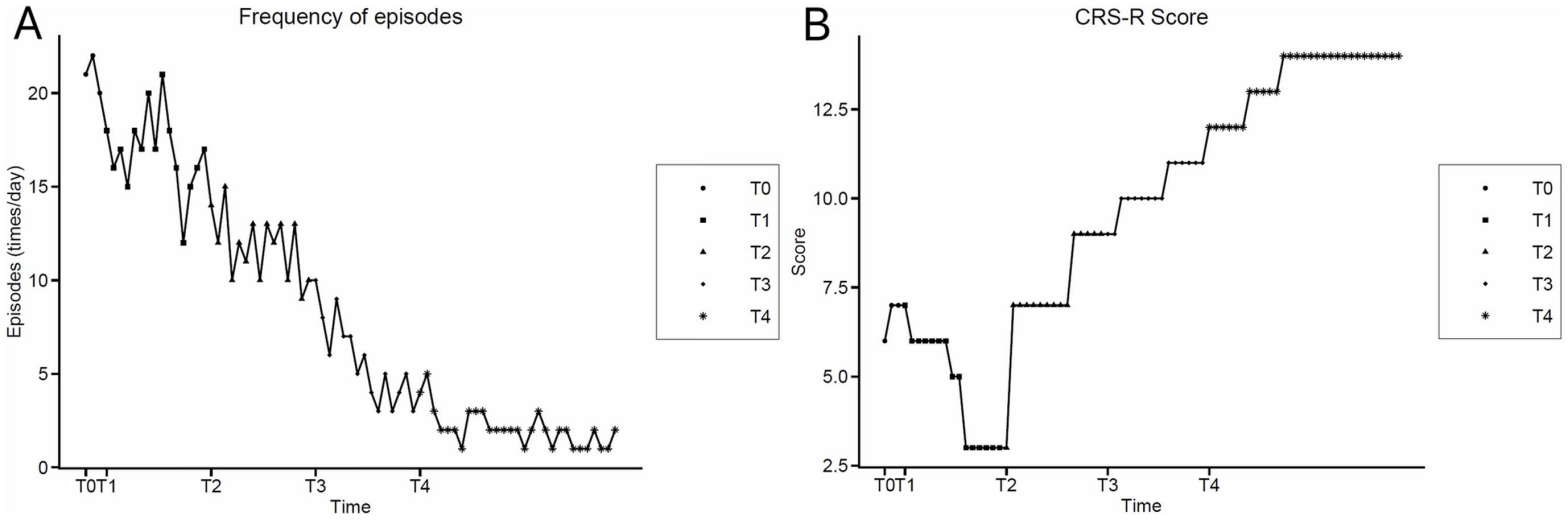
Trends of clinical indicators over time. **(A)** PSH episode frequency. ●: baseline (T0); ■: pharmacotherapy phase (T1); ▲: combined pharmacotherapy and taVNS phase (T2); ◆: taVNS consolidation with medication tapering (T3); ✳: final assessment after 8-week taVNS course (T4). **(B)** CRS-R score. Coma Recovery Scale–Revised scores assessed at corresponding time points.

After 4 weeks of taVNS treatment, PSH episode frequency showed a decreasing trend. Mild stimulation no longer triggered typical episodes. During episodes, tachypnea, tachycardia, hypertension, and increased muscle tone gradually improved. Consciousness level also improved, with the CRS-R score increasing to 9. Based on symptom improvement, baclofen and gabapentin were discontinued. Propranolol was reduced to 10 mg q8h for maintenance. After continued taVNS treatment for another 4 weeks (8 weeks total), only occasional mild tachypnea (26–33 breaths/min) occurred under strong stimulation (e.g., suctioning, patient transfer, and other high-intensity procedures) (Figure 3A), which resolved spontaneously without other sympathetic symptoms. Consciousness level further improved, with the CRS-R score increasing to 14 (Figure 3B). Vital signs and clinical assessments for each treatment phase are summarized in Table 2. During treatment, no obvious side effects of taVNS were observed (such as local skin reactions, pain, abnormal fluctuations in heart rate or blood pressure).

**Table 2:** Summary of vital signs and clinical assessments by treatment phase.

Phase	Duration	Pulse (bpm)	Respiratory rate (breaths/min)	BP systolic (mmHg)	BP diastolic (mmHg)	PSH episode frequency (/day)	CRS-R score
T0 (Baseline)	June 20–June 22	108–152	30–56	140–205	90–120	20–22	6–7
T1 (Pharmacotherapy)	June 23–July 7	90–148	14–53	128–190	78–120	12–21	3–7
T2 (taVNS + Pharmacotherapy)	July 8– July 22	90–139	22–52	121–180	69–110	9–15	3–9
T3 (taVNS consolidation + tapering)	July 23–August 6	82–128	16–38	110–158	69–112	3–10	9–11
T4 (Final assessment)	August 7–September 4	68–116	16–30	118–152	65–112	1–4	12–14

T0: baseline at PSH diagnosis (before pharmacotherapy). T1: after 2 weeks of pharmacotherapy (propranolol, baclofen, gabapentin, bromocriptine). T2: after 4 weeks of combined pharmacotherapy and taVNS (baclofen and gabapentin discontinued, propranolol reduced). T3: after 4 weeks of taVNS consolidation with medication tapering (propranolol 10 mg q8h). T4: final assessment after 8-week taVNS course. Values for pulse, respiratory rate, blood pressure, PSH episode frequency, and CRS-R scores are presented as range (min–max) observed during each phase. CRS-R, Coma Recovery Scale–Revised.

## Discussion

PSH is a common complication after severe brain injury. Its occurrence is associated with autonomic dysregulation resulting from an imbalance between central excitatory and inhibitory pathways (Scott and Rabinstein, 2020; Fnu et al., 2025). This report describes a patient with PSH following intracerebral hemorrhage. The pathophysiological process may have begun in the acute phase, but PSH was diagnosed after transfer to the neurorehabilitation department. Deep sedation and intensive blood pressure control in the neurosurgery intensive care unit may have masked typical PSH manifestations while stabilizing vital signs. After sedative agents were withdrawn and the treatment regimen was simplified, underlying autonomic imbalance became apparent, presenting as sympathetic episodes triggered by mild stimulation. A notable feature of this case was that after combination therapy with beta-blockers, central muscle relaxants, and GABAergic agents, heart rate, blood pressure, muscle tone, and sweating were controlled. However, tachypnea persisted and became the main problem affecting rehabilitation. After taVNS was added (Rebeiz et al., 2024; Zhang et al., 2025), refractory tachypnea gradually improved, and consciousness level also showed improvement (Zhou et al., 2023). It should be noted that in this case, “drug-refractory” specifically refers to the tachypnea symptom in PSH patients that responded poorly to conventional medications. This treatment outcome provides a new reference for the clinical management of PSH.

Combination pharmacotherapy is commonly used for PSH (Alison et al., 2024). Propranolol is used to control tachycardia and hypertension (Nguembu et al., 2021), baclofen to reduce muscle tone (Pucks-Faes et al., 2018), and gabapentin for sedation and anticonvulsant effects (Baguley et al., 2007). Initial treatment in this case showed that this regimen was effective for most symptoms. However, when tachypnea became an isolated and prominent symptom, clinical management became difficult. Deep sedation with dexmedetomidine and clonazepam controlled respiratory rate but decreased consciousness level (CRS-R decreased from 7 to 3) (Tu et al., 2021), which was inconsistent with the goal of arousal promotion in neurorehabilitation. This situation suggests that current medications have limitations in regulating respiratory centers. For such refractory PSH cases, interventions that can modulate specific neural circuits without affecting consciousness level need to be explored.

The vagus nerve is an important pathway connecting the brain and major organs. It participates in regulating heart rate, respiration, and digestion, and helps maintain internal homeostasis (Thompson et al., 2019). The core pathophysiology of PSH is sympathetic-parasympathetic imbalance, involving overactivation of sympathetic centers such as the hypothalamus and the rostral ventrolateral medulla (RVLM) and caudal ventrolateral medulla (CVLM) (Meyfroidt et al., 2017). TaVNS stimulates the auricular branch of the vagus nerve, enhances vagal afferent signals, and activates central nuclei such as the nucleus tractus solitarius (Frangos et al., 2015; Butt et al., 2020). It may further modulate the RVLM/CVLM and the hypothalamic-limbic system, potentially participating in descending inhibitory signals and influencing sympathetic output (Berthoud and Neuhuber, 2000). It should be noted that taVNS may also affect the norepinephrine and serotonin systems (Usher et al., 1999). Although these monoamines generally have excitatory effects, in specific neural circuits (e.g., by activating GABAergic inhibitory interneurons), their net effect may be stabilization rather than excitation. This may explain why taVNS could improve autonomic function without inducing excessive sympathetic excitation. In addition, the cholinergic anti-inflammatory pathway of taVNS may reduce post-brain-injury inflammation and its damage to the autonomic network (Lu et al., 2017; Jiang et al., 2015). This may have provided a favorable condition for the recovery of autonomic function in the present case. However, it should be noted that taVNS has a limited direct modulatory effect on the central respiratory rhythm generator, because respiratory control involves multiple non-vagal mechanisms, including the medullary respiratory center and the phrenic nerve. Clinical observation in this case showed that during taVNS treatment, baclofen and gabapentin were discontinued and propranolol dosage was reduced. This suggests that sympathetic overactivity may have improved. In addition, taVNS may activate ascending arousal pathways such as the locus coeruleus-norepinephrine system, enhance cortical excitability and network connectivity, and improve arousal and awareness levels (Usher et al., 1999). This may have implications for long-term neurological recovery and consciousness improvement (Poe et al., 2020; Alexis et al., 2025).

It is worth exploring to what extent the observed improvements can be attributed to taVNS rather than the effects of concurrent medication adjustments. In this case, refractory tachypnea persisted before taVNS initiation (after clonazepam and dexmedetomidine were discontinued) and showed gradual improvement after taVNS was started. Regarding consciousness level, the sustained increase in CRS-R scores was temporally synchronized with the 8-week taVNS course and occurred after sedative medications (clonazepam and dexmedetomidine) were withdrawn. These symptom improvements showed a temporal association with taVNS intervention. However, the influence of self-regulatory mechanisms following medication reduction cannot be completely excluded.

## Conclusion

This case suggests that for drug-refractory paroxysmal sympathetic hyperactivity, transcutaneous auricular vagus nerve stimulation may serve as a safe adjunctive intervention option. In this case, the application of taVNS showed a temporal association with tachypnea control and consciousness improvement, providing a preliminary reference for individualized treatment of PSH.

## Limitations

This study has several limitations. First, as a single case report, its generalizability is limited. The observed improvements are difficult to distinguish from the effects of spontaneous recovery after brain injury and concurrent medication adjustments. Second, objective indicators such as heart rate variability were not used to monitor changes in vagal tone, so mechanism discussion remains at a theoretical level. Third, although the stimulation parameters used in this case were based on previous literature, optimal stimulation site, treatment duration and frequency, long-term efficacy, and safety remain to be explored. These issues require validation through prospective studies. In addition, due to the limitations of the case report design, potential interactions between taVNS and medications (such as propranolol, baclofen, etc.) could not be evaluated. No obvious side effects were observed in this patient, but this finding cannot be generalized to other patients.

## Data Availability

The original contributions presented in the study are included in the article/supplementary material, further inquiries can be directed to the corresponding author.
